# The risk of cancer in HIV-infected people in southeast England: a cohort study

**DOI:** 10.1038/sj.bjc.6602273

**Published:** 2004-12-07

**Authors:** A Newnham, J Harris, H S Evans, B G Evans, H Møller

**Affiliations:** 1 Department of Primary Care and General Practice, The University of Birmingham, Edgbaston, Birmingham B15 2TT, UK; 2 CDSC HIV & STI Division, Health Protection Agency, Communicable Disease Surveillance Centre, 61 Colindale Avenue, Colindale, London NW9 5EQ, UK; 3 Thames Cancer Registry, Guy's, King's and St Thomas’ School of Medicine, 1st Floor, Capital House, 42 Weston Street, London SE1 3QD, UK

**Keywords:** HIV, AIDS, cohort study, linkage, England

## Abstract

This study used data from the Communicable Disease Surveillance Centre's national HIV database and the Thames Cancer Registry to assess the risk of cancer in HIV-infected people in southeast England. Among 26 080 HIV-infected men with 158 660 person-years follow-up, 1851 cancers, and among 7110 HIV-infected women (31 098 person-years), 171 cancers were identified. The standardised incidence ratio (SIR) for all non-AIDS-defining cancers was significantly increased in HIV-infected men (2.8, 95% confidence interval (CI) 2.6–3.1) but was nonsignificant in HIV-infected women (1.1, 95% CI 0.8–1.6). Most of the cancers observed were in men (*n*=1559) and women (*n*=127) with AIDS, and among them, the SIR for all non-AIDS-defining cancers was significantly increased in men (8.2, 95% CI 7.2–9.2) and women (2.8, 95% CI 1.6–4.6). The SIR for all non-AIDS-defining cancers was only just significantly increased in men with HIV-infection but not AIDS (1.2, 95% CI 1.0–1.5) and was nonsignificant in such women (0.8, 95% CI 0.5–1.2).

The epidemic of HIV-infection is continuing with nearly 6000 new HIV-infections reported in 2002, the highest annual total since 1985, and over 50 000 people reported with HIV-infection or AIDS in the UK by the end of 2001 (Health Protection Agency, 2004). People with HIV-infection are living longer due to the introduction of highly active antiretroviral therapies. Cancers and other illnesses that are related to HIV-infection may therefore become more common, and this may also be influenced by unknown long-term effects of the treatment.

By the end of 2001, nearly 70% of people reported to the Communicable Disease Surveillance Centre (CDSC) with HIV-infection or AIDS in the UK were reported from London and the Southeast. This corresponds approximately to the catchment area of the Thames Cancer Registry (TCR). The aim of this study was to link data from the cohort of people reported with HIV-infection or AIDS with data from the TCR to assess the risk of different types of cancer in HIV-infected people, those with AIDS and those with HIV-infection but not AIDS. In addition, we aimed to assess the completeness both of cancer registration in HIV-infected people and of HIV-infection reporting in people with cancer.

## MATERIALS AND METHODS

The CDSC collates information on the diagnosis of HIV-infection and AIDS in adults in England, Wales and Northern Ireland. Microbiologists and clinicians report demographic details and how the infection was probably acquired; the hospital trust from which the person was reported is also recorded. Names are not recorded but a soundex code is assigned to each record, comprising the first letter of the surname and three numbers based on the other letters. Soundex codes are not unique to one surname but can be used with other fields to detect duplicate entries. Deaths and AIDS diagnoses in previously reported people are also reported. The CDSC also receives information on HIV-infections and AIDS diagnoses in Scotland from the Scottish Centre for Infection and Environmental Health.

The TCR collects details of cancers diagnosed in southeast England in a catchment population of about 14 million people in London, Surrey, Sussex, Kent, Hertfordshire and Essex; it also records demographic data, including name, address and postcode. Patients are flagged in the NHS Central Register, which provides dates and causes of death. Cancer registration in England and the HIV/AIDS reporting system are both approved under the section 60 regulations of the Health and Social Care Act. The current study was approved by the Public Health Laboratory Service Ethics Committee and the West Midlands Multi-centre Research Ethics Committee.

The TCR data set in our study comprised the 224 199 cancers (ICD10 C00-97) that were diagnosed from 1985 to 2001 in people aged 15–59 years, who were resident in the TCR catchment area and had no cancer before 1985. The CDSC data set comprised the 50 182 HIV-infected people who were reported from the UK and diagnosed by the end of 2001. Both these data sets included date of birth, sex, soundex code, first initial and date of death. The TCR data also included second initial and details of the cancer. The CDSC data also included HIV-diagnosis date, whether the person had AIDS, AIDS-diagnosis date, when the person was last reported alive, and details of AIDS-defining cancers. A variable was created for whether the HIV-infection was first reported from a hospital in London, Southeast and Eastern regions, which includes the TCR catchment area. We then excluded people in the CDSC data whose first report was outside this area or who had no sex or date or birth, leaving cohorts of 27 054 men and 7510 women.

The two data sets were matched at the CDSC, accepting matched records if the date of birth, sex and soundex code were all identical. We excluded matches if the TCR date of death was before the date when the CDSC last knew that the person was alive, the dates of death differed by more than 30 days, or the CDSC date of death was more than 30 days before the cancer diagnosis date. We also excluded matches if the CDSC first initial did not match the first or second initial from the TCR database. Whereas the CDSC database is person-based, the TCR database is tumour-based. A tumour record should only match to one person, and we selected the best match for four tumour records that had each matched to two different people by reviewing the full patient records. A person could legitimately match to more than one tumour record, but we excluded second or third tumours to facilitate the calculation of person-years. This left 2139 one-to-one matches. We added the cancer diagnosis date, ICD10 three-digit cancer diagnosis code and TCR date of death to the CDSC data for people who had matched to a TCR tumour record.

To calculate person-years at risk, we created entry and exit dates for each person. For men and women with HIV-infection, we used whichever was later of the person's 15th birthday, their HIV-diagnosis date minus 30 days or 01/01/85, the start of the study period, as the entry date. For men and women with AIDS, we did the same but used the AIDS-diagnosis date minus 30 days. We backdated diagnosis dates so that cancers that may have initiated an HIV-diagnosis were included. We used the same exit date for men and women with HIV-infection and AIDS. We replaced 31/12/01, the end of the study period, with the CDSC date of death if this was present and before 31/12/01; with the TCR date of death if present and before 31/12/01; with the cancer diagnosis date if present; and with the person's 60th birthday if this was before the current exit date. We regarded the TCR dates of death as more accurate because they were supplied by the NHS Central Register.

Before each analysis, we excluded people with exit dates before 01/01/85, entry dates after 31/12/01, exit dates before entry dates, or the same entry and exit dates. The observed numbers of each cancer, all cancers and all non-AIDS-defining cancers were then counted for each cohort during the study period. AIDS-defining cancers are Kaposi's sarcoma (KS, ICD10 C46), non-Hodgkin's lymphoma (NHL, ICD10 C82-85) and cancer of the cervix uteri (ICD10 C53). For people with HIV-infection and AIDS, the person-years at risk in each sex and five-year age-band and period were multiplied by the incidence rate for each cancer site in southeast England during the same period. This gave an expected number of each cancer for each cohort during the study period. For the sexes separately, we subtracted the observed and expected numbers for each cancer in those with AIDS from the corresponding numbers for HIV-infected people to give the numbers for those with HIV-infection but not AIDS. The observed numbers of each cancer, all cancers and all non-AIDS-defining cancers were then divided by the expected number for each cohort and 95% confidence intervals (CI) calculated for the ratios.

Kaposi's sarcoma is very rare in young people without HIV-infection. To assess completeness of reporting of people with HIV-infection, we reviewed the proportion of KS in the TCR data that matched to a person in the CDSC data reported from anywhere in the UK. To assess completeness of cancer registration in HIV-infected people, we reviewed the proportion of people from the CDSC data with both an AIDS-defining cancer and a first report from regions wholly or partly within the TCR catchment area who matched to a comparable tumour record from the TCR data. We compared presumptive or definitive KS and Burkitt's lymphoma or immunoblastic lymphoma from the CDSC data with, respectively, KS and NHL from the TCR data. Burkitt's lymphoma and immunoblastic lymphoma are classified as NHL by the TCR.

## RESULTS

### HIV-infection

After making the exclusions based on entry and exit dates, the analysis included 26 080 HIV-infected men who contributed 158 660 person-years and 1851 cancers. The SIR for all cancers was 11.5 (95% CI 10.9–12.0), and the SIR for non-AIDS-defining cancers was 2.8 (95% CI 2.6–3.1) (Table 1). The SIR was significantly decreased for cutaneous malignant melanoma (CMM), and significantly increased for 13 cancer sites: anus and anal canal, liver, bronchus and lung, other skin, KS, nerves and soft tissue, secondary lymph node, secondary other sites, site unknown, Hodgkin's disease, NHL, leukaemias, and other lymphoid haematopoietic. The highest SIR was for KS (237, 95% CI 221–253).

The analysis included 7110 HIV-infected women who contributed 31 098 person-years and 171 cancers. The SIR for all cancers (5.0, 95% CI 4.2–5.8) was lower than in HIV-infected men, and the SIR for non-AIDS-defining cancers (1.1, 95% CI 0.8–1.6) was nonsignificant (Table 1). There was no cancer site where the SIR was significantly decreased. In contrast to HIV-infected men, the SIR was significantly increased at only four cancer sites: KS, secondary lymph node, NHL, and other lymphoid haematopoietic. The SIR for KS (774; 95% CI 589–999) was, however, higher in women than men with HIV-infection.

### AIDS

After making the exclusions based on entry and exit dates, this analysis included 10 522 men with AIDS who contributed 30 221 person-years to the analysis. Nearly 85% (*n*=1559) of the observed cancers in HIV-infected men were in men with AIDS. The SIR for all cancers was 42.3 (95% CI 40.2–44.5), and for non-AIDS-defining cancers 8.2 (95% CI 7.2–9.2) (Table 2). The SIR was significantly increased for 14 cancer sites: anus and anal canal, liver, bronchus and lung, other skin, KS, nerves and soft tissue, penis, secondary lymph node, secondary other sites, site unknown, Hodgkin's disease, NHL, leukaemias, and other lymphoid haematopoietic. There was no cancer site where the SIR was significantly decreased. The highest SIR was for KS (1082, 95% CI 1008–1160).

This analysis included 1604 women with AIDS who contributed 4728 person-years to the analysis. Nearly 75% (*n*=127) of the observed cancers in HIV-infected women were in women with AIDS. The SIR for all cancers (21.0, 95% CI 17.5–25.0) was lower than in men with AIDS, as was the SIR for non-AIDS-defining cancers (2.8, 95% CI 1.6–4.6) (Table 2). There were no cancer sites where the SIR was significantly decreased. In contrast to men with AIDS, the SIR was significantly increased at only five cancer sites: other skin, KS, secondary lymph node, NHL, and other lymphoid haematopoietic. The SIR for KS (4356, 95% CI 3263–5698) was, however, higher in women than men with AIDS.

### HIV-infection but not AIDS

Men with HIV-infection but not AIDS contributed 128 438 person-years and 292 cancers to the analysis. The SIR for all cancers was 2.3 (95% CI 2.1–2.6), but for non-AIDS-defining cancers the increase was only marginal (1.2, 95% CI 1.0–1.5) (Table 3). The SIR was significantly increased for seven cancer sites: anus and anal canal, liver, other skin, KS, Hodgkin's disease, NHL, and other lymphoid haematopoietic. For multiple myeloma, the SIR was increased but the confidence interval was very close to nonsignificance. The SIR was significantly decreased for CMM. The highest SIR was for other lymphoid haematopoietic cancer (29.3, 95% CI 12.7–57.8).

Women with HIV-infection but not AIDS contributed 26 370 person-years and 44 cancers. The SIR for all cancers was 1.5 and only just significantly increased (95% CI 1.1–2.1), and the SIR for non-AIDS-defining cancers was only 0.8 (95% CI 0.5–1.2) (Table 3). The SIR was significantly increased for three cancer sites: KS, NHL, and other lymphoid haematopoietic. There was no site where the SIR was significantly decreased. The highest SIR was for KS (93.7, 95% CI 34.4–204).

### Completeness of ascertainment

There were 1251 cases of KS in the TCR data and 1017 (81%) had a valid match to a person in the CDSC data reported from anywhere in the UK. This included 11 matches where KS was the second or third tumour. Matched cases (94%, *n*=954) were more likely than unmatched cases (88%, *n*=207) to be in men. The matched group was also younger than the unmatched group: for example, 94% (*n*=960) of the matched group were aged under 50 years compared with 87% (*n*=203) of the unmatched group. Matched cases were also more likely to have died by the end of the study period (69%, *n*=699) than unmatched cases (44%, *n*=103).

There were 2079 people from the CDSC data with presumptive or definitive KS and a first report from a region located wholly or partly within the TCR catchment area and 648 (31%) had a valid match to a KS record in the TCR data. This included six matches where the KS from the TCR data was the second or third tumour. There were 516 people from the CDSC data with Burkitt's lymphoma or immunoblastic lymphoma and a first report from a region located wholly or partly within the TCR catchment area, and 228 (44%) had a valid match to an NHL record from the TCR data. This included matches involving two cases of NHL from the TCR data that were second or third tumours.

## DISCUSSION

This study used data from the national database of HIV-infected people and a regional cancer registry to assess the risk of cancer in HIV-infected people in the TCR area, which has the highest number of people reported with HIV-infection in England. Despite including about 33 000 HIV-infected people, there were few cancers in women. Although much smaller than certain linkage studies of people with AIDS (Goedert *et al*, 1998; Frisch *et al*, 2001; Gallagher *et al*, 2001; Mbulaiteye *et al*, 2003), our cohorts were larger than other studies of HIV-infected people (Cooksley *et al*, 1999; Grulich *et al*, 2002; Allardice *et al*, 2003).

False matches would mean that observed cancers were overestimated, whereas too few true matches would mean the reverse. Assessing the accuracy of matching is difficult because there is no gold standard and it is subject to the degree of completeness of the databases. Nearly 20% of KS from the TCR data did not match to a person reported to the CDSC from anywhere in the UK. Assuming that these tumours were correctly classified as KS, they could be in people without HIV-infection, HIV-infected people who were reported to the CDSC but that we failed to match with their tumour record, or HIV-infected people who were not reported to the CDSC. The first explanation is unlikely as KS in younger people without HIV-infection is very rare. The second explanation is possible, and we would have underestimated observed cancers and, therefore, SIRs if this were the case. The third explanation is also possible. Under-reporting for AIDS has been estimated to be about 10% (Evans, 1995), and the TCR may be aware of cases of which the CDSC is unaware because the CDSC and the TCR obtain details of cases via separate mechanisms. Staff from the TCR retrieve information from patient notes, whereas the CDSC relies on reports from clinicians. Any incompleteness of reporting of HIV-infected people would mean that we underestimated observed and expected cancers.

We matched less than one-third of people from the CDSC data with presumptive or definitive KS and a first report from a region located wholly or partly within the TCR catchment area to a TCR KS record. This suggests incompleteness of registration of KS by the TCR. The case definition of KS used by the CDSC and the TCR are similar, but sexually transmitted infection clinic notes may not be available to staff who are registering cancers, which could lead to diagnoses made in this setting being missed by the cancer registry. Incompleteness of cancer registration may vary by site and be higher for KS than other sites. We matched a higher, but still fairly low, proportion (44%) of people from the CDSC data with Burkitt's lymphoma or immunoblastic lymphoma and a first report from a region located wholly or partly within the TCR catchment area to a TCR NHL record. The overall completeness of registrations 5 years after diagnosis has been estimated as 92% (Bullard *et al*, 2000), but failure to register cancers may be more likely in HIV-infected people than the general population. Overall, any incompleteness of cancer registration would mean that we underestimated observed cancers and SIRs.

During the overall analyses and the assessment of the completeness of cancer registration in HIV-infected people, we excluded people from the CDSC data whose first report was not from a region located wholly or partly within the TCR catchment area, that is not from London, Southeast and Eastern Regions. As the Southeast and Eastern Regions extend beyond the TCR catchment area, the cohorts will have included people from outside the TCR catchment area. Some of the apparent incompleteness of registration of KS and NHL from the CDSC data by the TCR may, therefore, be explained by their registration at other regional cancer registries. Overall, the inclusion of people from outside the TCR catchment area will have meant that person-years at risk and expected cancers were overestimated and SIRs were underestimated.

To examine the robustness of our estimates, we created different entry and exit dates. The SIRs and profile of cancers with significantly increased or decreased SIRs were generally similar with entry dates created using the HIV- or AIDS-diagnosis date minus 30 days or the HIV- or AIDS-diagnosis date itself.

Two other exit dates were created by using the CDSC last known alive date instead of the TCR and CDSC dates of death, and by omitting the steps involving the dates of death and only using the cancer diagnosis date and the person's 60th birthday. Observed cancers were the same for each exit date because the penultimate step was always to replace the current date with the cancer diagnosis date if present, but total person-years at risk and, therefore, expected cancers and SIRs varied. Expected cancers were generally highest and SIRs were generally lowest when exit dates were based only on cancer diagnosis dates and 60th birthdays. For HIV-infected men, expected cancers were not consistently lower for exit dates based on last known alive dates or dates of death. For HIV-infected women, expected cancers were generally lowest and SIRs were generally highest for exit dates based on last known alive dates. For men and women with AIDS, expected cancers were generally lowest and SIRs were generally highest for exit dates based on dates of death rather than last known alive dates. This was because a smaller proportion of people with AIDS than all HIV-infected people had a last known alive date. Although it seemed reasonable to use exit dates based on dates of death for this study, it may be more accurate to combine last known alive dates and dates of death in a single exit date in future analyses.

Comparing our results with results from other studies is complicated by different methods and time periods used. Results from other studies were also usually presented for men and women combined. The introduction of highly active antiretroviral therapy in 1996 may also have altered the risk of some cancers. It has been suggested that people may cease to be at risk of KS once immune function has been improved by therapy (Ledergerber *et al*, 1999), and there was a significant decrease in the risk of KS and NHL but not other cancers from 1992–1996 to 1997–1999 when data from 23 prospective studies were combined (International Collaboration on HIV and Cancer, 2000). Conversely, people may now survive longer to develop a cancer associated with other risk factors. The risk of lung cancer increased between 1992–1995 and 1996–1999 in a cohort of about 77 000 HIV-infected men identified through French hospitals (Herida *et al*, 2003).

Studies have been largely consistent for people with AIDS. We observed an increased risk of lung cancer, Hodgkin's disease and all non-AIDS-defining cancers in people with AIDS in southeast England compared with the general population. This was consistent with studies linking cancer registry data with data from people who were diagnosed with AIDS in the USA from 1978 to 1996 (Frisch *et al*, 2001) and Italy from 1985 to 1998 (Dal Maso *et al*, 2003), and people who were notified up to 1999 to the Australian HIV and AIDS databases (Grulich *et al*, 2002). We also observed an increased risk of anal cancer and leukaemia consistent with the studies from Italy and the USA, an increased risk of cancer of nerves and soft tissues consistent with the studies from Australia and the USA, and an increased risk of liver cancer and cancer of the oral cavity consistent with the study from the USA. We found an increased risk of penile cancer in men with AIDS consistent with the study from the USA, but we only observed two such cancers.

Results for non-AIDS-defining cancers for HIV-infected people were not as consistent. Even so, we found an increased risk of liver cancer, lung cancer and all non-AIDS-defining cancers in HIV-infected people, which was consistent with a Scottish study linking cancer registry data with data from people with HIV-infection and/or AIDS from 1981 to 1996 (Allardice *et al*, 2003). We also found an increased risk of anal cancer, cancer of nerves and soft tissues, Hodgkin's disease, leukaemia and all non-AIDS-defining cancers consistent with an increased risk of these cancers in HIV-infected people in Australia (Grulich *et al*, 2002). The increased risk of Hodgkin's disease was also consistent with an increased risk in HIV-infected men in Texas that was observed when cancer registry data from 1975 to 1994 were linked with data from people with HIV-infection and/or AIDS from 1981 to 1994 (Cooksley *et al*, 1999).

The increased risk of other skin cancer in HIV-infected people was higher in this study than other studies (Cooksley *et al*, 1999; Allardice *et al*, 2003). This may suggest that some non-KS skin cancers in this study were actually KS. We also found that HIV-infected men had a decreased risk of CMM. This could indicate that HIV-infection protects against CMM but may be more likely to indicate that CMMs were under ascertained or misclassified as KS. Any resulting increase in the number of KS was probably balanced by KS that were wrongly assigned as other skin cancers. Misclassification of Hodgkin's disease and NHL may also partly explain the increased risk of other lymphoid haematopoietic cancer in men and women with HIV-infection, AIDS, and HIV-infection but not AIDS in this study.

We also assessed the risk of cancer in HIV-infected people who had not had an AIDS diagnosis reported. The excess of KS and NHL in these men and women is an artefact. If the matches were correct, the people involved should have been reported with AIDS and not included in this part of the analysis, although any other cancers in these people were excluded when we excluded second and third tumours. Most of the increased risk in HIV-infected people in this study was related to AIDS, which suggests that, so far, most cancers in HIV-infected people have been associated with AIDS-related immunosuppression. The risk only remained increased for anal cancer, liver cancer, other skin cancer, Hodgkin's disease, and other lymphoid haematopoietic cancer, and the risk of all non-AIDS-defining cancers was only just increased in HIV-infected men without AIDS. The increased risk of liver cancer may have been due to the comparison of cohorts of people with a high and low prevalence of hepatitis B infection. Chronic infection with hepatitis B causes liver cancer. Prevalence is much higher in Africa than Western Europe (International Agency for Research on Cancer, 1994), and over 70% of new HIV-diagnoses in the UK ascribed to heterosexual transmission in 2001 were in people from, or who had acquired their infection in, Africa (Communicable Disease Surveillance Centre, 2002).

Other groups assessed the risk of cancer before AIDS by linking data from cancer registries and AIDS registers and examining periods from 5 years to 6 months before AIDS (Frisch *et al*, 2001; Grulich *et al*, 2002; Dal Maso *et al*, 2003). The risk of anal cancer and Hodgkin's disease was increased before AIDS in Italy and Australia, similar to this study, and the risk of all non-AIDS-defining cancers was increased in Italy (Grulich *et al*, 2002; Dal Maso *et al*, 2003). It has, however, been suggested that this method will under ascertain pre-AIDS cancers because some HIV-infected people with cancer will not survive to AIDS (Engels *et al*, 2002). In Australia, when the analysis included people with HIV-infection who had not progressed to AIDS as well as people with AIDS from the date of their HIV-infection to 5 years before AIDS, the risk of liver cancer was increased similar to this study (Grulich *et al*, 2002). In Australia, cancer registry data were also linked with data from people from the National HIV Database who were followed to 6 months before AIDS, cancer, death, or the end of the study period (Li *et al*, 2002), but only anal and testicular cancer had a significantly increased risk.

This study has established the feasibility of assessing the risk of cancer in HIV-infected adults using data from the CDSC and a UK regional cancer registry. Factors that could have affected the estimates of observed and expected cancers have been identified and should be further explored in any future analyses. Future analyses could also involve stratification of the cohort by subgroups based on route of acquisition, ethnicity or country of birth as well as examine different periods to explore possible effects on cancer risk of highly active anti-retroviral therapy. This will also facilitate comparison with the results of studies from other time periods.

## Figures and Tables

**Table 1:** Standardised incidence ratios for cancers (ICD10 C00-97) in men and women with HIV-infection in southeast England. Cancer sites with no observed cancers in men or women are not included in the table

	**Men**				**Women**				**All**			
	**Obs**	**SIR**	**LCI**	**UCI**	**Obs**	**SIR**	**LCI**	**UCI**	**Obs**	**SIR**	**LCI**	**UCI**
Oral (C01–10,14)	5	1.0	0.3	2.3	1	2.5	0.1	14.0	6	1.1	0.4	2.3
Nasopharynx (C11)	3	4.1	0.8	11.9	1	17.1	0.4	95.2	**4**	**5.0**	**1.4**	**12.8**
Oesophagus (C15)	2	0.5	0.1	1.8	0	0.0			2	0.5	0.1	1.8
Stomach (C16)	2	0.4	0.1	1.6	0	0.0			2	0.4	0.1	1.5
Small intestine (C17)	2	3.8	0.5	13.6	0	0.0			2	3.4	0.4	12.4
Colorectal (C18–20)	12	0.8	0.4	1.4	2	1.5	0.2	5.4	14	0.9	0.5	1.5
Anus and anal canal (C21)	**17**	**25.1**	**14.6**	**40.2**	1	9.7	0.2	53.9	**18**	**23.1**	**13.7**	**36.5**
Liver (C22)	**13**	**5.9**	**3.1**	**10.1**	0	0.0			**13**	**5.6**	**3.0**	**9.6**
Pancreas (C25)	3	0.8	0.2	2.4	0	0.0			3	0.8	0.2	2.3
Other digestive (C26)	1	3.3	0.1	18.1	0	0.0			1	3.1	0.1	17.0
Nasal cavity (C30,31)	1	1.8	0.05	10.1	0	0.0			1	1.7	0.04	9.4
Larynx (C32)	5	2.1	0.7	4.9	0	0.0			5	2.0	0.7	4.8
Bronchus, lung (C33,34)	**38**	**2.3**	**1.6**	**3.2**	1	1.0	0.02	5.4	**39**	**2.2**	**1.6**	**3.1**
Thymus, heart (C37,38)	1	1.7	0.04	9.4	0	0.0			1	1.6	0.04	8.7
Bone (C40,41)	1	1.0	0.03	5.5	0	0.0			1	0.9	0.02	4.9
Skin melanoma (C43)	**2**	**0.2**	**0.03**	**0.8**	0	0.0			**2**	**0.2**	**0.02**	**0.6**
Other skin malignant (C44)	**68**	**20.6**	**16.0**	**26.2**	2	7.2	0.9	26.1	**70**	**19.6**	**15.3**	**24.8**
Kaposi's sarcoma (C46)	**872**	**236.5**	**221.1**	**252.7**	**59**	**774.2**	**589.4**	**998.7**	**931**	**247.4**	**231.8**	**263.8**
Nerves, soft tissue (C47,49)	**6**	**3.6**	**1.3**	**7.9**	0	0.0			**6**	**3.1**	**1.2**	**6.8**
Breast (C50)					12	0.8	0.4	1.4	12	0.8	0.4	1.4
Cervix uteri (C53)					3	1.0	0.2	2.9	3	1.0	0.2	2.9
Ovary (C56)					2	1.0	0.1	3.7	2	1.0	0.1	3.7
Penis (C60)	3	3.9	0.8	11.5					3	3.9	0.8	11.5
Prostate (C61)	5	0.9	0.3	2.0					5	0.9	0.3	2.0
Testis (C62)	19	1.1	0.6	1.7					19	1.1	0.6	1.7
Kidney (C64,65)	6	1.2	0.4	2.6	0	0.0			6	1.1	0.4	2.5
Bladder (C67)	3	0.5	0.1	1.5	0	0.0			3	0.5	0.1	1.5
Meninges, brain and spinal cord (C70–72)	9	1.1	0.5	2.0	0	0.0			9	1.0	0.4	1.8
Thyroid (C73)	1	0.7	0.02	3.6	0	0.0			1	0.4	0.01	2.3
Secondary lymph node (C77)	**31**	**34.0**	**23.1**	**48.3**	**3**	**35.0**	**7.2**	**102.1**	**34**	**34.1**	**23.6**	**47.6**
Secondary respiratory/digestive (C78)	2	1.1	0.1	4.1	0	0.0			2	1.0	0.1	3.7
Secondary other sites (C79)	**8**	**6.5**	**2.8**	**12.9**	0	0.0			**8**	**6.0**	**2.6**	**11.9**
Site unknown (C80)	**54**	**17.4**	**13.0**	**22.6**	1	2.9	0.1	16.4	**55**	**15.9**	**12.0**	**20.7**
Hodgkin's disease (C81)	**36**	**6.1**	**4.3**	**8.5**	2	2.3	0.3	8.3	**38**	**5.6**	**4.0**	**7.7**
Non-Hodgkin's lymphoma (C82–85)	**571**	**40.9**	**37.7**	**44.4**	**75**	**61.5**	**48.4**	**77.1**	**646**	**42.6**	**39.4**	**46.0**
Multiple myeloma (C90)	5	2.4	0.8	5.6	1	7.5	0.2	41.7	6	2.7	1.0	5.9
Leukaemias (C91–95)	**18**	**2.6**	**1.6**	**4.2**	1	1.4	0.03	7.6	**19**	**2.5**	**1.5**	**3.9**
Other lymphoid, haematopoietic (C96)	**26**	**75.5**	**49.3**	**110.6**	**4**	**173.7**	**47.3**	**444.7**	**30**	**81.6**	**55.1**	**116.5**
												
All non-AIDS-defining cancers	**408**	**2.8**	**2.6**	**3.1**	34	1.1	0.8	1.6	**442**	**2.5**	**2.3**	**2.8**
All cancers	**1851**	**11.5**	**10.9**	**12.0**	**171**	**5.0**	**4.2**	**5.8**	**2022**	**10.3**	**9.9**	**10.8**

Bold values indicate cancer sites where the 95% confidence interval does not include one.

**Table 2:** Standardised incidence ratios for cancers (ICD10 C00-97) in men and women with AIDS in southeast England. Cancer sites with no observed cancers in men or women are not included in the table

	**Men**				**Women**				**All**			
	**Obs**	**SIR**	**LCI**	**UCI**	**Obs**	**SIR**	**LCI**	**UCI**	**Obs**	**SIR**	**LCI**	**UCI**
Oral (C01–10,14)	4	3.2	0.9	8.1	1	17.0	0.4	95.0	**5**	**3.8**	**1.2**	**8.8**
Nasopharynx (C11)	1	6.2	0.2	34.5	0	0.0			1	5.8	0.1	32.5
Oesophagus (C15)	1	1.0	0.02	5.5	0	0.0			1	1.0	0.02	5.3
Stomach (C16)	2	1.8	0.2	6.6	0	0.0			2	1.8	0.2	6.3
Small intestine (C17)	1	8.0	0.2	44.7	0	0.0			1	7.4	0.2	41.3
Colorectal (C18–20)	5	1.4	0.4	3.2	1	4.0	0.1	22.4	6	1.5	0.6	3.4
Anus and anal canal (C21)	**7**	**42.8**	**17.2**	**88.1**	0	0.0			**7**	**38.1**	**15.3**	**78.5**
Liver (C22)	**4**	**7.6**	**2.1**	**19.5**	0	0.0			**4**	**7.4**	**2.0**	**18.8**
Pancreas (C25)	2	2.2	0.3	8.0	0	0.0			2	2.1	0.3	7.7
Other digestive (C26)	1	13.4	0.3	74.4	0				1	13.4	0.3	74.4
Nasal cavity (C30,31)	1	7.9	0.2	44.0	0				1	7.9	0.2	44.0
Larynx (C32)	2	3.3	0.4	11.8	0	0.0			2	3.2	0.4	11.6
Bronchus, lung (C33,34)	**25**	**6.0**	**3.9**	**8.9**	1	5.0	0.1	27.8	**26**	**6.0**	**3.9**	**8.8**
Thymus, heart (C37,38)	1	7.9	0.2	43.8	0				1	7.9	0.2	43.8
Bone (C40,41)	1	5.3	0.1	29.3	0	0.0			1	4.8	0.1	26.5
Skin melanoma (C43)	1	0.5	0.01	2.9	0	0.0			1	0.4	0.01	2.4
Other skin malignant (C44)	**55**	**71.3**	**53.7**	**92.8**	**2**	**41.6**	**5.1**	**150.3**	**57**	**69.5**	**52.7**	**90.1**
Kaposi's sarcoma (C46)	**787**	**1082.0**	**1007.7**	**1160.3**	**53**	**4356.0**	**3262.9**	**5697.7**	**840**	**1135.9**	**1060.3**	**1215.3**
Nerves, soft tissue (C47,49)	**5**	**14.9**	**4.9**	**34.9**	0	0.0			**5**	**13.4**	**4.3**	**31.2**
Breast (C50)					1	0.4	0.01	2.0	1	0.4	0.01	2.0
Cervix uteri (C53)					1	2.1	0.1	11.4	1	2.1	0.1	11.4
Penis (C60)	**2**	**11.1**	**1.3**	**40.0**					**2**	**11.1**	**1.3**	**40.0**
Prostate (C61)	1	0.6	0.02	3.4					1	0.6	0.02	3.4
Testis (C62)	6	1.9	0.7	4.0					6	1.9	0.7	4.0
Kidney (C64,65)	2	1.7	0.2	6.0	0	0.0			2	1.6	0.2	5.8
Bladder (C67)	1	0.7	0.02	4.0	0	0.0			1	0.7	0.02	3.9
Meninges, brain and spinal cord (C70–72)	5	2.8	0.9	6.5	0	0.0			5	2.6	0.8	6.0
Secondary lymph node (C77)	**29**	**135.4**	**90.7**	**194.4**	**3**	**195.7**	**40.4**	**571.7**	**32**	**139.4**	**95.4**	**196.8**
Secondary respiratory/digestive (C78)	2	4.7	0.6	16.8	0	0.0			2	4.3	0.5	15.4
Secondary other sites (C79)	**6**	**20.3**	**7.4**	**44.2**	0	0.0			**6**	**19.0**	**7.0**	**41.4**
Site unknown (C80)	**51**	**68.4**	**50.9**	**89.9**	1	16.0	0.4	89.1	**52**	**64.3**	**48.0**	**84.4**
Hodgkin's disease (C81)	**16**	**15.0**	**8.6**	**24.4**	0	0.0			**16**	**13.4**	**7.7**	**21.7**
Non-Hodgkin's lymphoma (C82–85)	**502**	**163.5**	**149.5**	**178.4**	**58**	**279.8**	**212.5**	**361.7**	**560**	**170.8**	**157.0**	**185.6**
Multiple myeloma (C90)	0	0.0			1	38.4	1.0	214.1	1	1.8	0.05	10.2
Leukaemias (C91–95)	**12**	**8.1**	**4.2**	**14.2**	1	9.0	0.2	50.1	**13**	**8.2**	**4.4**	**14.0**
Other lymphoid, haematopoietic (C96)	**18**	**250.2**	**148.3**	**395.4**	**3**	**820.1**	**169.1**	**2396.1**	**21**	**277.8**	**172.0**	**424.6**
												
All non-AIDS-defining cancers	**270**	**8.2**	**7.2**	**9.2**	**15**	**2.8**	**1.6**	**4.6**	**285**	**7.4**	**6.6**	**8.3**
All cancers	**1559**	**42.3**	**40.2**	**44.5**	**127**	**21.0**	**17.5**	**25.0**	**1686**	**39.3**	**37.4**	**41.2**

Bold values indicate cancer sites where the 95% confidence interval does not include one.

**Table 3:** Standardised incidence ratios for cancers (ICD10 C00-97) in men and women with HIV-infection but not AIDS in southeast England. Cancer sites with no observed cancers in men or women are not included in the table

	**Men**				**Women**				**All**			
	**Obs**	**SIR**	**LCI**	**UCI**	**Obs**	**SIR**	**LCI**	**UCI**	**Obs**	**SIR**	**LCI**	**UCI**
Oral (C01–10,14)	1	0.3	0.01	1.4	0	0.0			1	0.2	0.01	1.3
Nasopharynx (C11)	2	3.5	0.4	12.5	1	20.6	0.5	114.8	3	4.8	1.0	14.0
Oesophagus (C15)	1	0.3	0.01	1.9	0	0.0			1	0.3	0.01	1.8
Small intestine (C17)	1	2.5	0.1	13.7	0	0.0			1	2.2	0.1	12.5
Colorectal (C18–20)	7	0.6	0.3	1.3	1	0.9	0.02	5.1	8	0.7	0.3	1.3
Anus and anal canal (C21)	**10**	**19.5**	**9.3**	**35.8**	1	12.0	0.3	66.8	**11**	**18.4**	**9.2**	**33.0**
Liver (C22)	**9**	**5.3**	**2.4**	**10.1**	0	0.0			**9**	**5.1**	**2.3**	**9.7**
Pancreas (C25)	1	0.4	0.01	2.1	0	0.0			1	0.3	0.01	1.9
Larynx (C32)	3	1.7	0.3	4.9	0	0.0			3	1.6	0.3	4.8
Bronchus, lung (C33,34)	13	1.1	0.6	1.8	0	0.0			13	1.0	0.5	1.7
Skin melanoma (C43)	**1**	**0.1**	**0.004**	**0.8**	0	0.0			**1**	**0.1**	**0.003**	**0.6**
Other skin malignant (C44)	**13**	**5.2**	**2.7**	**8.8**	0	0.0			**13**	**4.7**	**2.5**	**8.1**
Kaposi's sarcoma (C46)	**85**	**28.7**	**22.9**	**35.5**	**6**	**93.7**	**34.4**	**203.9**	**91**	**30.1**	**24.2**	**36.9**
Nerves, soft tissue (C47,49)	1	0.8	0.02	4.2	0	0.0			1	0.7	0.02	3.6
Breast (C50)					11	0.9	0.4	1.6	11	0.9	0.4	1.6
Cervix uteri (C53)					2	0.8	0.1	2.9	2	0.8	0.1	2.9
Ovary (C56)					2	1.2	0.2	4.5	2	1.2	0.2	4.5
Penis (C60)	1	1.7	0.04	9.5					1	1.7	0.04	9.5
Prostate (C61)	4	0.9	0.3	2.4					4	0.9	0.3	2.4
Testis (C62)	13	0.9	0.5	1.5					13	0.9	0.5	1.5
Kidney (C64,65)	4	1.1	0.3	2.7	0	0.0			4	1.0	0.3	2.5
Bladder (C67)	2	0.5	0.1	1.6	0	0.0			2	0.4	0.1	1.6
Meninges, brain and spinal cord (C70–72)	4	0.6	0.2	1.5	0	0.0			4	0.5	0.1	1.4
Thyroid (C73)	1	0.8	0.02	4.6	0	0.0			1	0.5	0.01	2.8
Secondary lymph node (C77)	2	2.9	0.3	10.4	0	0.0			2	2.6	0.3	9.4
Secondary other sites (C79)	2	2.2	0.3	7.8	0	0.0			2	2.0	0.2	7.2
Site unknown (C80)	3	1.3	0.3	3.7	0	0.0			3	1.1	0.2	3.3
Hodgkin's disease (C81)	**20**	**4.2**	**2.5**	**6.4**	2	2.7	0.3	9.8	**22**	**4.0**	**2.5**	**6.0**
Non-Hodgkin's lymphoma (C82–85)	**69**	**6.3**	**4.9**	**8.0**	**17**	**16.8**	**9.8**	**26.9**	**86**	**7.2**	**5.8**	**8.9**
Multiple myeloma (C90)	**5**	**3.2**	**1.0**	**7.4**	0	0.0			5	3.0	1.0	6.9
Leukaemias (C91–95)	6	1.1	0.4	2.4	0	0.0			6	1.0	0.4	2.2
Other lymphoid, haematopoietic (C96)	**8**	**29.3**	**12.7**	**57.8**	**1**	**51.6**	**1.3**	**287.7**	**9**	**30.8**	**14.1**	**58.5**
												
All non-AIDS-defining cancers	**138**	**1.2**	**1.0**	**1.5**	19	0.8	0.5	1.2	157	1.2	1.0	1.4
All cancers	**292**	**2.3**	**2.1**	**2.6**	**44**	**1.5**	**1.1**	**2.1**	**336**	**2.2**	**2.0**	**2.4**

Bold values indicate cancer sites where the 95% confidence interval does not include one.
